# Target-Controlled  Infusion for Caesarean Delivery Under General Anesthesia: From Conventional Pharmacokinetic Models to Physiologically Based Pharmacokinetic Modeling

**DOI:** 10.3390/life16050739

**Published:** 2026-04-29

**Authors:** Matild Keresztes, Leonard Azamfirei, Emoke Almasy, Janos Szederjesi

**Affiliations:** 1 Doctoral School of Medicine and Pharmacy, George Emil Palade University of Medicine, Pharmacy, Science and Technology of Târgu Mures, 540139 Târgu Mureș, Romania; keresztes.matild.25@stud.umfst.ro; 2 Department of Anesthesiology and Intensive Care, George Emil Palade University of Medicine, Pharmacy, Science and Technology of Târgu Mureș, 540139 Târgu Mureș, Romania; leonard.azamfirei@umfst.ro (L.A.); yangzi37@gmail.com (J.S.)

**Keywords:** TIVA, TCI, obstetric anesthesia, PBPK modeling

## Abstract

Target-controlled infusion (TCI) enables the precise delivery of intravenous anesthetics based on pharmacokinetic–pharmacodynamic (PK–PD) models and represents a key component of total intravenous anesthesia (TIVA). However, its use in obstetric anesthesia remains limited, as current TCI algorithms are derived from non-pregnant populations and do not account for pregnancy-related physiological changes or maternal–fetal drug distribution. This narrative review examines the clinical application of TIVA-TCI in caesarean delivery under general anesthesia, summarizing evidence from recent observational studies and national audits, which suggest feasibility but limited adoption in routine obstetric practice. Pregnancy induces significant alterations in drug distribution, protein binding, metabolism, and clearance, which may affect anesthetic pharmacokinetics and fetal exposure. Physiologically based pharmacokinetic (PBPK) modeling is explored as a complementary approach that may improve understanding of maternal–fetal drug disposition by integrating physiological and drug-specific parameters. Although promising, these model-based strategies require further validation before clinical implementation. Overall, current evidence supports the cautious use of TIVA-TCI in selected obstetric settings while highlighting the need for pregnancy-specific pharmacokinetic models and prospective clinical studies.

## 1. Introduction

Target-controlled infusion (TCI) is a drug delivery system that uses continuous intravenous drug administration, in which a microprocessor calculates the rate of drug infusion based on a target plasma or effect-site concentration set by the anesthetist [1,2]. These microprocessor-driven systems incorporate different pharmacokinetic (PK) and pharmacodynamic (PD) models for intravenous anesthetics [3,4] and opioids [5], thereby revolutionizing the practice of total intravenous anesthesia (TIVA) [6,7]. Because real-time plasma or effect-site drug concentrations are not measured in routine clinical practice, PK/PD algorithms estimate drug concentrations based on patient-specific covariates [1,8,9,10].

In obstetric practice, emergencies may arise from maternal factors, fetal factors, or a combination of both [11,12,13]. Depending on the degree of urgency and the required decision-to-delivery interval, obstetric emergencies are commonly classified into four categories, reflecting increasing levels of maternal and/or fetal risk and guiding the timing and conduct of anesthetic and obstetric interventions [14]. In addition to obstetric indications, anesthesia-related emergencies may also necessitate urgent intervention, including failed or inadequate neuraxial anesthesia or complications associated with general anesthesia, such as accidental awareness during rapid sequence induction [15].

Although neuraxial anesthesia is the gold standard technique for caesarean delivery, there are clinical situations in which general anesthesia is required [16], and in such cases, TIVA-TCI may represent a viable anesthetic option [17,18,19].

This study was conducted as a narrative review to summarize and critically evaluate current evidence on target-controlled infusion for caesarean delivery under general anesthesia, with a focus on the transition from conventional pharmacokinetic models to physiologically based pharmacokinetic modeling. This review aims to bridge the gap by combining clinical evidence on TIVA-TCI use in caesarean delivery with a mechanistic analysis of pregnancy-related pharmacokinetic alterations and evaluating PBPK modeling as a framework for developing pregnancy-adapted TCI strategies.

This review is novel not because it is the first to discuss total intravenous anesthesia for caesarean delivery, but because it brings together three evidence streams that are usually discussed separately: contemporary obstetric TIVA-TCI practice, pregnancy-related determinants of maternal–fetal anesthetic disposition, and the translational potential of maternal–fetal PBPK modeling. Its main contribution is therefore a framework for distinguishing evidence-based clinical conclusions from mechanistic or model-based inferences.

## 2. Methodology

A literature search was performed using PubMed/Scopus with combinations of the following keywords: “target-controlled infusion” or “TIVA” + “caesarean delivery”. The search was limited to clinical studies, reviews, and relevant publications from the past 10 years, while earlier landmark studies were included when appropriate. Relevant studies, including clinical investigations, multicenter evaluations, national audits, and international guidelines, were selected based on clinical relevance and scientific importance. A total of 19 articles were identified. The reference list was further expanded to include pharmacological models relevant to pregnancy.

As a narrative review, no predefined inclusion or exclusion criteria or formal assessment of study quality was applied. The selected literature was analyzed thematically, focusing on pharmacokinetic changes during pregnancy, limitations of current TCI models, and the potential role of PBPK modeling in obstetric anesthesia.

Because direct obstetric evidence for propofol-based TIVA-TCI remains limited, this review distinguishes between three evidence categories: direct obstetric clinical data; pregnancy physiology and placental-transfer evidence; and model-based or in silico inference. Conclusions are framed according to the strongest supporting evidence available.

**Figure 1:**
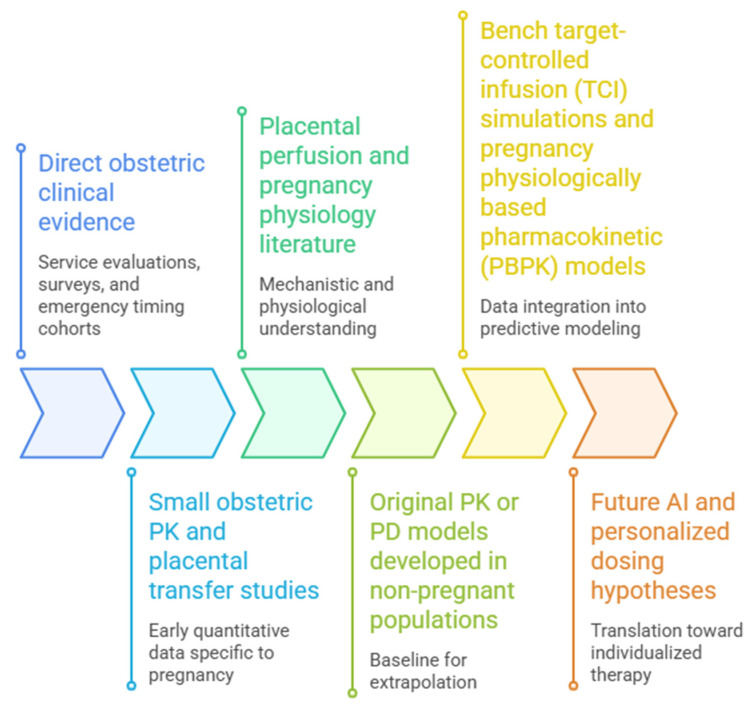
Illustration of a conceptual workflow of this narrative review, for integrating evidence to inform pharmacological modeling and personalized dosing in pregnancy. The process begins with direct obstetric clinical evidence, including service evaluations, surveys, and emergency timing cohorts. This is complemented by small obstetric pharmacokinetic (PK) and placental transfer studies providing early quantitative data. Mechanistic insight is added through the placental perfusion and pregnancy physiology literature. These data are integrated with original PK or pharmacodynamic (PD) models developed in non-pregnant populations, serving as a baseline for extrapolation. The combined evidence informs bench target-controlled infusion (TCI) simulations and pregnancy physiologically based pharmacokinetic (PBPK) models, ultimately leading to future applications in artificial intelligence and personalized dosing strategies.

For better understanding, please refer to Figure 1.

The figures included in this manuscript were created by the authors using Illustrae, a scientific illustration tool.

## 3. TIVA-TCI Use in Caesarean Delivery

### 3.1. Clinical Guidelines for TIVA

In 2019, A. F. Nimmo et al. published comprehensive joint guidelines from the Association of Anesthetists and the Society for Intravenous Anesthesia on the safe practice of TIVA in the general (non-obstetric) population [20]. These guidelines do not recommend the routine use of total intravenous anesthesia in obstetric anesthesia; however, they state that if TIVA is employed, the principles outlined in the guidelines should be strictly followed. While the recommendations primarily refer to TIVA as a general anesthetic technique, they specify that when general anesthesia is maintained with propofol infusion, the use of a TCI system is recommended.

The Chinese Clinical Practice Guidelines for TIVA, published in late 2025, provide a valuable international perspective. They are structured in 22 recommendations spanning 12 clinical issues. Within the obstetric context, the guidelines advocate for the use of TIVA-TCI, particularly for managing hypertensive disorders. Additionally, the consensus states that processed EEG (pEEG), such as the Bispectral Index (BIS), is strongly recommended for every patient undergoing TIVA to ensure safety and monitor depth of anesthesia [21].

### 3.2. Clinical Evidence and Real-World Use

In non-obstetric settings, TIVA improves the quality of recovery by significantly reducing postoperative nausea and vomiting [22]. Additionally, it offers a superior environmental profile by eliminating the use of halogenated volatile gases, which are potent contributors to greenhouse gas emissions [23].

While documented in obstetrics, TIVA use remains largely confined to small case series and high-risk indications like maternal cardiac, neurology, and muscular diseases [19,22,24,25,26,27,28,29,30,31,32].

Beyond isolated case reports, multicenter service evaluations and national surveys now provide the most robust evidence regarding the real-world application of TIVA-TCI in obstetric anesthesia. These investigations mark a transition from theoretical feasibility to established clinical practice. The main characteristics and findings of recent studies and guidelines on TIVA use in obstetric anesthesia are summarized in Table 1.

A landmark contribution in this area is the 2024 ObsTIVA-UK project, a prospective evaluation that scrutinized 123 cases (6.6% of obstetric general anesthesia) across 30 UK maternity units (16% of total maternity units) to characterize the contemporary landscape of intravenous techniques in the delivery suite. In this evaluation, most caesarean deliveries (63%) were elective. The survey revealed that, although TIVA is technically feasible for all categories of urgency, it is currently most prevalent in elective settings, with 99% of clinicians utilizing TCI rather than manual dosing. Despite the availability of modern pharmacokinetic models, the study indicated clinical reliance on the Marsh (62%) and Schnider (35%) models for propofol and the Minto (100%) model for remifentanil, with the physiologically more appropriate Eleveld model for propofol accounting for only 3% of cases. The evaluation suggested a favorable maternal recovery profile but noted a high incidence of transient neonatal respiratory depression, with 57% of infants requiring initial support, though all achieved Apgar scores > 7 by the 10-min mark. A limitation of the current data is that the total duration of TIVA neonatal exposure was not documented [17].

The NAP7 Activity Survey provides ” vit’l national context for TIVA use, reinforcing the “niche” status found in the ObsTIVA-UK study but highlighting a broader shift in UK practice. Across all surgical specialties, TIVA use has seen a dramatic increase, rising from 8% in 2013 (NAP5) to 26% in 2021 (NAP7). NAP7 identifies that the obstetric population is becoming increasingly high-risk, with a higher prevalence of obesity (average BMI increasing from 24.9 to 26.7) and comorbidities. The survey highlights that obstetric anesthesia/analgesia represents 55% of all anesthetic activity overnight [33]. Since TIVA in obstetrics is typically reserved for daytime, consultant-led elective cases, the “out-of-hours” nature of the specialty remains a major barrier to its routine adoption.

### 3.3. Time-Critical Considerations in Emergency Caesarean Delivery

In obstetric anesthesia, timeliness is commonly evaluated using the decision-to-delivery interval (DDI), with a widely adopted benchmark of ≤30 min for Category 1 Caesarean section. However, the DDI comprises several clinically relevant sub-intervals, including the time required to establish anesthesia, the interval to skin incision, and the incision-to-delivery time.

Dunn et al. (2016) conducted a retrospective cohort study evaluating operational timings and clinical outcomes in Category 1 caesarean delivery within a tertiary obstetric unit operating a dedicated “crash caesarean section” protocol [34]. The primary focus was on the assessment of the DDI and its association with perinatal outcome measures. A total of 390 Category 1 caesarean deliveries were analyzed. The service achieved a mean DDI of 9.4 ± 3.2 min, and all cases met the ≤30-min benchmark commonly used for Category 1 caesarean delivery. General anesthesia was employed in most cases (88.9%), reflecting its role in facilitating expedited delivery under urgent conditions. In stratified analyses comparing DDI < 10 min versus ≥10 min, a shorter DDI was not associated with statistically significant improvements in perinatal outcomes.

McCahon and Catling (2003) performed a retrospective review comparing the time required to achieve surgical readiness for emergency caesarean section under general anesthesia (GA) versus spinal anesthesia [35]. Surgical readiness was defined as the point at which the patient was prepared for skin incision, incorporating anesthetic establishment and necessary pre-incision preparations. Data from 137 emergency caesarean deliveries were analyzed. The mean time to surgical readiness was significantly shorter with GA than with spinal anesthesia (15.4 min [range 2–44] vs. 27.6 min [range 13–55], respectively; *p* < 0.01). The authors concluded that GA facilitates more rapid progression to incision readiness in emergency caesarean delivery.

In obstetric anesthesia, the interval between maternal tracheal intubation and umbilical cord clamping represents a critical window of vulnerability for neonatal drug exposure. During this phase, highly lipophilic agents like propofol and remifentanil rapidly traverse the placental barrier. A recent simulation study by Osthoff et al. (2025) [36] revealed a significant clinical paradox: in 40–50% of all caesarean deliveries—and a striking 62% of emergency cases—the fetus is delivered during a TCI ‘infusion pause.’ This pause represents a mechanical artifact of traditional three-compartment models, such as the Schnider model, which suspend drug delivery after the induction bolus to allow plasma concentrations to equilibrate with lower maintenance targets. Consequently, neonatal exposure is not a result of steady-state infusion but is almost exclusively driven by the kinetics of the initial maternal bolus and its rapid redistribution into the fetal–placental unit.

Claims regarding the use of TIVA-TCI in emergency caesarean delivery should be interpreted cautiously. Here, the evidence base consists of emergency obstetric timing studies, general TIVA safety guidance, and recent bench simulation, rather than comparative obstetric outcome trials.

### 3.4. Practical Limitations of TIVA-TCI in Emergency Settings

Although rapid sequence induction in obstetric anesthesia is typically performed using intravenous agents, the use of total intravenous anesthesia for maintenance remains limited in emergency caesarean delivery. This is primarily due to the need for rapid anesthetic establishment, the perceived risk of intraoperative awareness, and the logistical complexity associated with target-controlled infusion systems. In addition, uncertainty regarding pharmacokinetic model accuracy in pregnant patients and concerns about fetal drug exposure further limit its adoption in time-critical situations.

When TCI propofol is used for induction, a high initial target concentration can be selected to simulate a bolus dose through rapid infusion, followed by reduction in the target concentration once the desired effect is achieved. However, compared with manual bolus administration, TCI-based induction may result in slower time to loss of consciousness, which can be suboptimal in emergency settings. This limitation may be partially mitigated by co-administration of fast-acting opioids such as remifentanil or alfentanil. In addition, if the induction dose is administered manually while using a TCI pump for maintenance, the predicted plasma concentrations displayed by the device may be inaccurate during the early phase of anesthesia [20].

For rapid sequence induction, intravenous agents are routinely used: in healthy pregnant women, propofol (1.5–2.5 mg/kg) combined with rocuronium (0.6–1.0 mg/kg) is commonly administered, whereas in patients with hemodynamic instability or poor tolerance to hemodynamic fluctuations, etomidate (0.2–0.3 mg/kg) may be preferred [21].

It is important to acknowledge that the current evidence base for TIVA-TCI use in obstetric anesthesia remains limited. Most available data are derived from observational studies, audits, and extrapolation from non-obstetric populations, while robust prospective studies specifically conducted in pregnant patients are scarce. In addition, pharmacokinetic models used in clinical practice have not been adequately validated in this population. These limitations should be considered when interpreting the available evidence and highlighting the need for further research in this field.

## 4. Maternal Gestational Adaptation: Impacts on the Pharmacokinetics of Lipophilic Anesthetic Agents

Currently used TCI algorithms were derived from non-obstetric adult populations, and their application in parturients represents an extrapolation that may not fully account for pregnancy-related pharmacokinetic and pharmacodynamic changes (Table 2). Accordingly, statements regarding dosing accuracy in pregnancy should be interpreted as concerns about external validity rather than as proof of model failure.

**Figure 2:**
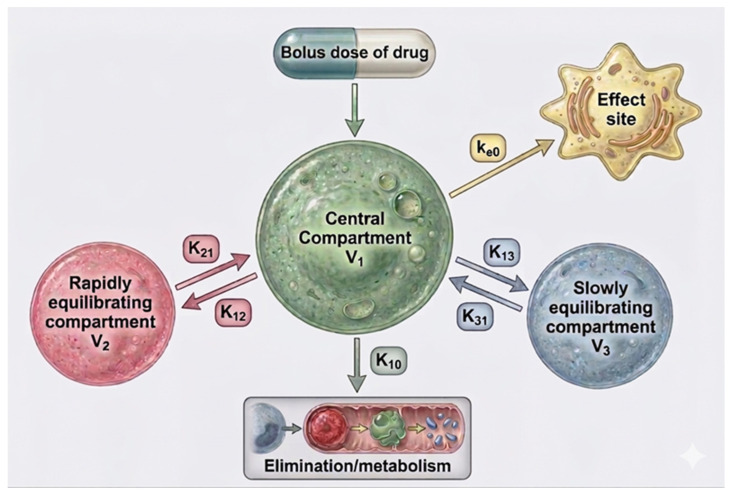
Conventional three-compartment pharmacokinetic model illustrating drug distribution between the central and peripheral compartments and the effect site. This model forms the basis of commonly used TCI algorithms and represents a simplified, theoretical framework derived from non-pregnant populations.

These algorithms are based on a three-compartment pharmacokinetic model. A bolus dose refers to a single intravenous administration directly into the bloodstream. The central compartment (V_1_) represents blood and highly perfused organs (e.g., brain, heart, and lungs), where the drug is initially distributed. The rapidly equilibrating compartment (V_2_) includes tissues with fast drug exchange (e.g., liver and kidneys), while the slowly equilibrating compartment (V_3_) represents tissues with slower uptake and release (e.g., muscle and fat). The effect site corresponds to the location of pharmacological action, with equilibration governed by the rate constant k_e0_. Intercompartmental transfer is described by rate constants k_12_/k_21_ (V_1_ ↔ V_2_) and k_13_/k_31_ (V_1_ ↔ V_3_), while k_10_ represents drug elimination from the central compartment (Figure 2).

Pregnancy is associated with well-defined physiological alterations, including an increase in plasma volume of approximately 40–50%, an increase in cardiac output of 30–50%, and an increase in glomerular filtration rate of up to 50%. In parallel, plasma protein concentrations, particularly albumin, are reduced due to hemodilution.

These pregnancy-related changes provide a strong mechanistic basis for altered an aesthetic disposition, but the quantitative implications for obstetric TCI dosing remain incompletely defined because modern anesthetic PK studies in pregnant patients are sparse.

**Figure 3:**
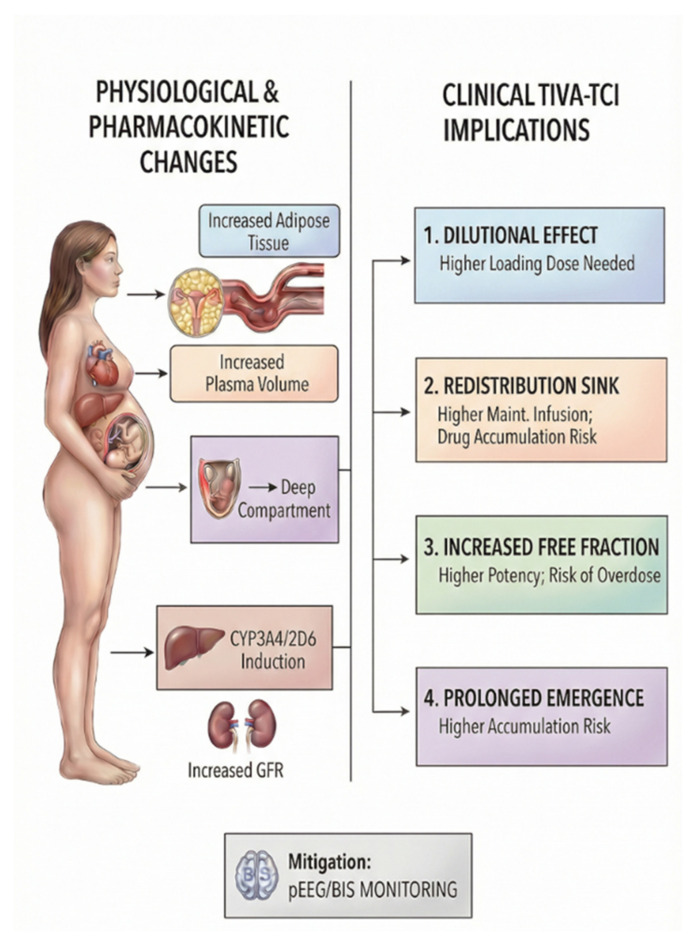
Physiological changes during pregnancy influencing the pharmacokinetics of lipophilic anesthetic agents, illustrating proposed mechanisms based on current physiological and pharmacokinetic understanding. Pregnancy is associated with expansion of central and peripheral distribution compartments, the presence of the feto-placental unit as an additional compartment, induction of selected cytochrome P450 enzymes, and increased glomerular filtration rate. These changes may lead to dilutional effects, enhanced redistribution, an increased free drug fraction due to reduced protein binding, and potential prolongation of drug elimination and emergence.

Theoretically, these changes influence drug distribution, metabolism, elimination, and cardiovascular function, necessitating careful consideration when administering anesthetics to pregnant patients (Figure 3) [37].

### 4.1. Alterations in Distribution Dynamics

Pregnancy is associated with marked expansion of both central and peripheral distribution spaces, increasing the volume of distribution of lipophilic agents [38,39]. Maternal plasma volume rises by ~40–50%, enlarging the central compartment and potentially attenuating peak plasma concentrations after a standard bolus, which may reduce early effect-site levels [40]. In parallel, increased maternal adiposity expands peripheral drug storage for highly lipophilic agents such as propofol, potentially prolonging redistribution and context-sensitive decline after infusion cessation [41,42]. Finally, the feto-placental unit introduces an additional “deep” compartment: although placental transfer is rapid, fetal tissues and amniotic fluid may function as a secondary reservoir, complicating washout kinetics during emergence [42,43,44]. The Marsh model, which uses total body weight as the primary covariate, may overestimate central volume in parturients with gestational weight gain, whereas the Schnider model, incorporating lean body mass and age, may better approximate distribution but still lacks pregnancy-specific adjustments. More recent population models, such as Eleveld, which integrate broader covariates and allometric scaling, may provide improved predictions across heterogeneous populations; however, these models have not been specifically validated in pregnant patients.

### 4.2. Hemodilution and Protein Binding Kinetics

Gestational hemodilution reduces circulating concentrations of key drug-binding proteins [45,46]. Maternal albumin and α1-acid glycoprotein levels fall as plasma volume expansion outpaces protein synthesis, thereby potentially decreasing available binding sites. For lipophilic drugs such as propofol and several opioids, this shift increases the unbound (pharmacologically active) fraction, potentially enhancing clinical effect at a given total plasma concentration [47]. Consequently, because standard TCI algorithms are generally parameterized using total drug concentrations in non-obstetric populations, careful titration to clinical endpoints—ideally supported by processed EEG monitoring—may be required to avoid unintended relative overdosing in parturient [42].

### 4.3. Metabolic and Clearance Shifts

During pregnancy, the clearance of intravenous anesthetic agents is modified by combined changes in regional perfusion and gestation-related alterations in metabolic capacity. For high-extraction drugs (e.g., propofol), clearance is largely perfusion-limited; therefore, enhanced hepatic blood flow may increase systemic clearance and contribute to higher maintenance infusion requirements. In contrast, elimination of low-extraction drugs is more dependent on intrinsic enzymatic activity, making clearance particularly sensitive to pregnancy-related changes in drug-metabolizing enzymes [48,49].

Gestational increases in estrogen and progesterone can induce selected cytochrome P450 isoenzymes, notably CYP3A4 and CYP2D6, which may accelerate the biotransformation of commonly used sedatives and opioids (e.g., midazolam and fentanyl) and thereby shorten their effective duration [50].

Renal handling also changes substantially in pregnancy. A physiological rise in renal plasma flow and glomerular filtration rate—often approaching a 50% increase—occurs early in gestation, and emerging data suggest upregulation of specific renal transporters, further enhancing tubular secretion. Although most lipophilic TIVA agents are primarily eliminated by hepatic metabolism, their hydrophilic metabolites may be cleared more efficiently in pregnancy, potentially reducing the likelihood of metabolite accumulation compared with the non-pregnant state [49,51].

## 5. Placental Transfer and Fetal Drug Exposure of Lipophilic Anesthetic Agents

Placental drug transfer depends on factors such as lipophilicity, molecular weight, protein binding, and placental blood flow, which generally favor the transfer of small, lipophilic anesthetic agents across the placenta [44].

Propofol is highly lipophilic and therefore rapidly crosses the placental barrier, producing detectable concentrations in the fetal circulation shortly after maternal administration. Clinical studies in caesarean delivery have reported umbilical venous concentrations approximately 22–32% of maternal levels, indicating significant but partial fetal exposure. Despite this transfer, neonatal outcomes are generally favorable, with most infants demonstrating normal Apgar scores and rapid postnatal recovery, suggesting that the neonatal effects of propofol are usually transient [19].

Early clinical studies by Dailland et al., Gin et al., and Sánchez-Alcaraz et al. demonstrated that propofol reaches the fetal circulation within minutes after maternal induction, with maternal concentrations generally exceeding umbilical venous concentrations, and umbilical arterial concentrations lower still, suggesting ongoing fetal redistribution after transfer [52,53,54].

Experimental work in human placental models suggests that fetal albumin can modulate the extent of transfer, which may help explain why fetal exposure is significant but still somewhat buffered relative to maternal plasma levels [55].

Remifentanil, a µ-opioid receptor agonist with extremely rapid metabolism by nonspecific plasma and tissue esterases, also crosses the placenta readily because of its lipophilic properties.

In a randomized clinical study, Hu et al. evaluated placental transfer of propofol and remifentanil during caesarean section under general anesthesia. Both drugs were detected in maternal arterial and umbilical cord blood at delivery, confirming transplacental passage. However, fetal concentrations remained lower than maternal levels, and no significant differences in Apgar scores or neonatal neurological adaptation were observed between short and prolonged induction-to-delivery intervals [56].

More recent pharmacokinetic studies have further demonstrated measurable concentrations of remifentanil in maternal arterial, umbilical venous, and umbilical arterial blood during caesarean delivery, confirming transplacental passage but suggesting limited accumulation in the fetus due to rapid hydrolysis and redistribution [57].

## 6. In Silico Simulations and Physiologically Based Pharmacokinetics Model (PBPK)

Traditionally, the pharmacokinetics of a chemical compound are determined through pre-clinical animal studies and, for promising candidates, clinical trials in humans. However, these approaches are costly, labor-and resource-intensive, time-consuming, and unsuitable for screening large numbers of drug candidates. These limitations can become even more pronounced in obstetric patients, where ethical constraints, safety concerns, and the need to protect both mother and fetus further restrict the feasibility of extensive in vivo experimentation. PBPK modeling addresses this need by integrating physiological and physicochemical data to simulate a compound’s movement and performance in vivo [58,59].

**Figure 4:**
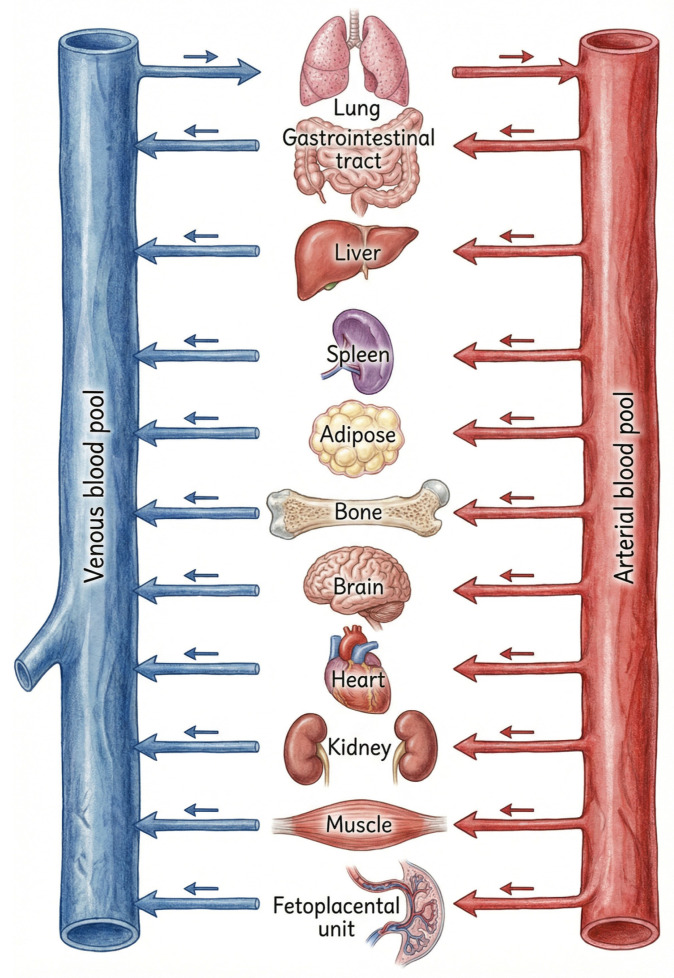
Physiologically based pharmacokinetic (PBPK) model illustrating drug distribution across maternal organs and the feto-placental unit. Unlike conventional compartmental models, PBPK incorporates physiological parameters such as organ blood flow, tissue volume, and protein binding, enabling simulation of pregnancy-specific drug disposition.

A physiologically based pharmacokinetic model is a mechanistic framework used to describe and predict the absorption, distribution, metabolism, and excretion (ADME) of a drug by explicitly incorporating human anatomy and physiology. In PBPK modelling, the body is represented as interconnected compartments that correspond to real organs and tissues, linked by empirically derived blood flow rates (Figure 4) [58,60]. Drug movement between compartments is governed by parameters such as tissue–plasma partition coefficients, protein binding, and membrane permeability, while elimination is modelled using organ-specific processes [59,60].

Compared with traditional compartmental pharmacokinetic models, PBPK approaches have greater physiological interpretability and allow simulation of drug exposure under changing biological conditions [61]. They differ from standard TCI models because they use actual anatomical and physiological parameters rather than abstract “compartments” [60].

PBPK model inputs are derived from in vitro experiments, in vivo preclinical studies, and clinical data, and the model is iteratively refined as additional in vitro and in vivo information becomes available. Depending on system complexity, both drug-dependent and organ-specific parameters can be adjusted to represent virtually any physiological or clinical condition [58,62].

This is particularly relevant in pregnancy, where alterations in cardiac output, plasma volume, body composition, renal function, and metabolic enzyme activity—and the presence of a feto-placental unit—can substantially modify maternal and fetal drug concentrations [59,63,64].

**Figure 5:**
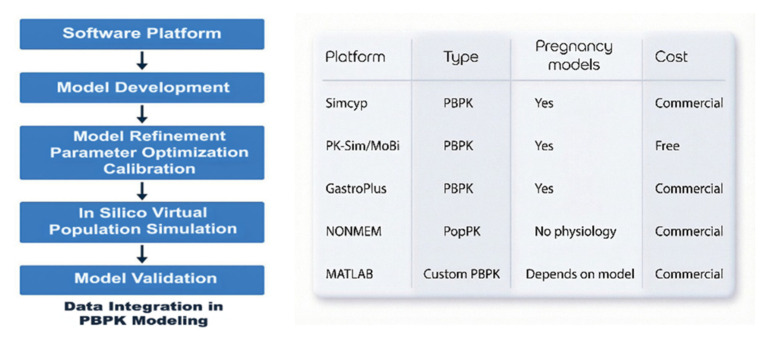
Data integration steps for PBPK modelling (**left**). Schematic comparison of major pharmacokinetic and PBPK simulation platforms used in pharmacological research (**right**). PBPK: physiology-based pharmacokinetic, PopPK: population pharmacokinetic.

Several simulation platforms are available for physiologically based pharmacokinetic modeling in pregnancy (Figure 5). Commercial systems such as the Simcyp Simulator and GastroPlus incorporate validated pregnancy populations and maternal–fetal transfer models [65,66,67,68,69,70,71]. Open-source alternatives including PK-Sim and MoBi provide flexible PBPK frameworks allowing custom maternal–placental–fetal model construction [69,72]. In addition, pharmacometrics platforms such as NONMEM (nonlinear mixed-effects modeling) enable population pharmacokinetic analysis and Monte Carlo simulation, although they lack explicit physiological structure [73]. These modeling tools are increasingly applied to predict maternal drug exposure, placental transfer, and fetal drug concentrations in obstetric anesthesia [74].

A recent PBPK modelling study by Le Merdy et al. developed a maternal–fetal pharmacokinetic framework incorporating pregnancy-related physiological changes. The model was applied to several metabolized drugs, including midazolam, metoprolol, metronidazole and successfully predicted maternal and fetal drug exposure across different stages of pregnancy. The model was first calibrated using clinical pharmacokinetic data from healthy non-pregnant volunteers. After validation, the model was used to simulate drug exposure in pregnant women and fetuses at different gestational stages. The predicted maternal concentrations were compared with published clinical pharmacokinetic data in pregnant women, and the model was able to reproduce the differences caused by pregnancy. The PBPK framework developed by Le Merdy et al. was implemented using the GastroPlus platform [67].

PBPK modeling is best regarded, at present, as a translational and hypothesis-generating framework for obstetric anesthesia. While pregnancy PBPK models have shown value in maternal–fetal pharmacology, their application to propofol/remifentanil dosing in caesarean delivery requires compound-specific qualification and prospective clinical validation before clinical implementation.

## 7. Future Perspectives: Precision TIVA in Obstetric Anesthesia

Despite advances in target-controlled infusion technology, currently available pharmacokinetic models are derived from non-pregnant populations and may not adequately reflect pregnancy-specific physiology. Future research should focus on the development and validation of models that incorporate maternal–fetal physiological changes.

Physiologically based pharmacokinetic modeling represents a promising approach to support this process by enabling simulation of drug exposure under pregnancy-specific conditions. In the future, integration of such models into clinical decision-making tools may allow more individualized anesthetic dosing [75,76].

In parallel, advances in computational medicine may enable the use of machine-learning algorithms to refine anesthetic dosing strategies. These approaches could incorporate large clinical datasets, pharmacokinetic simulations, and intraoperative monitoring data to optimize drug delivery in real time [72,77,78,79]. Ultimately, the integration of PBPK modeling, advanced computational methods, and individualized patient data may facilitate the development of personalized obstetric anesthesia, improving both maternal safety and neonatal outcomes [80].

## 8. Conclusions

The principal contribution of this review is not to provide validated pregnancy-specific dosing recommendations but to clarify where current obstetric TIVA-TCI practice is supported by direct evidence and where future advances will depend on mechanistic and model-informed research.

These formulations fit the current evidence base because recent practice data in the United Kingdom remain observational, pregnancy PK evidence is limited, and the PBPK literature is stronger as a regulatory and translational framework than as a clinically validated obstetric anesthesia tool.

Target-controlled infusion represents an important advancement in total intravenous anesthesia; however, its application in obstetric anesthesia remains limited. Current TCI models are derived from non-pregnant populations and may not adequately account for pregnancy-related physiological changes or maternal–fetal drug distribution.

Physiologically based pharmacokinetic modeling provides a promising framework to better understand these alterations and support the development of pregnancy-adapted approaches. However, further research and clinical validation are required before such strategies can be routinely implemented in obstetric practice.

## Figures and Tables

**Table 1:** Summary of recent studies and guidelines addressing the use of total intravenous anesthesia (TIVA) in obstetric anesthesia. CD: caesarean delivery, PONV: postoperative nausea and vomiting, PPH: postpartum haemorrhage. GA: general anesthesia. TCI: target controlled infusion.

Study/Guideline	ObsTIVA-UK Research Report	NAP7 Activity Survey	Chinese Clinical Practice Guidelines for TIVA
Author (Year)	Metodiev et al. (2024) [17]	Andrew D. Kane et al. (2023) [33]	Yi Feng et al. (2025) [21]
Type of Study	Multicenter observational project/registry	National audit, snapshot activity survey (UK)	National clinical practice guideline
Data collection interval	November 2022 and June 2023	8–24 November 2021. Recorded details of all surgical cases undertaken over 4 days	2024
No. of Cases	30 maternity units 90,782 births → 37,281 (41%) CD 1877 GA for obstetric surgical procedures (not limited to CDs) 123 (6.6%) utilized TIVA: 104 + 19 104 patients who underwent CD under TIVA 19 patients had TIVA, reasons other than CD	352 hospitals completed the survey → 24,172 cases included → 1681 (7%) CD	Not applicable (guideline synthesis). 21 experts → total of 22 recommendations were made on 12 clinical issues
Indications for TIVA	Neurological disorder (13%), malignant hyperthermia (1%), cardiac disorder (4%), anesthetist preference (84%), severe PONV (1%), risk of PPH (34%)	Across all surgical specialties, TIVA use has seen a dramatic increase, rising from 8% in 2013 (NAP5) to 26% in 2021. Reason: environmental impact, focus on better recovery profiles, proposed benefits for cancer recurrence, increasing equipment availability, technique embedded within the new UK postgraduate curriculum	Recommendation 1: malignant hyperthermia, intracranial hypertension, long QT syndrome Recommendation 11.2: Propofol-based TIVA can be applicable in CD. In hypertensive disorders, remifentanil as induction agent can be used, accompanied by preparations for neonatal resuscitation
TIVA delivery method	99% TCI 1% Manual Infusion	No data collected	Recommendation 6: Drugs with shorter context-sensitive half-times are indicated for TCI. Propofol- or remifentanil-based TCI is recommended
Maternal Outcomes	Reduced PONV Median recorded blood loss during CD 600 mL (438–1000 mL)	No data collected	Recommendation 2.1: TIVA can reduce the incidence of PONV
Neonatal Outcomes	57% required some form of respiratory support. Apgar scores < 7 at 1 min 73%. Apgar scores improved at 5 and 10 min.	No data collection	Neonatal depression can occur if continuous i.v. propofol infusion dose exceeds 9.0 mg/kg/h

**Table 2:** Comparison of commonly used pharmacokinetic models in target-controlled infusion. V: volume (compartment), k: constants.

Model	Marsh (1991)	Schnider (1998)	Eleveld (2018)	Minto (1997)
Drug	Propofol	Propofol	Propofol	Remifentanil
Targeting Mode	Plasma concentration (Cp)	Plasma (Cp) and effect-site (Ce)	Plasma (Cp) and effect-site (Ce)	Plasma (Cp) and effect-site (Ce)
Covariates	Total body weight	Age, gender, weight, height, lean body mass (LBM)–James equation	Age, weight, gender, height, comedication (opioid), fat-free mass	Age, weight, lean body mass
Central compartment	V1	V1	V1	V1
Peripheral compartments	V2, V3	V2, V3	V2, V3	V2, V3
Elimination/transfer constants	k10, k12, k21, k13, k31	k10, k12, k21, k13, k31	k10, k12, k21, k13, k31	k10, k12, k21, k13, k31
Effect-site parameter	Not included in original model; Added in modified Marsh as k_e0_ 0.26 min^−1^ → 1.2 min^−1^	k_e0_ = 0.456 min^−1^	k_e0_	k_e0_
Main parameters adjusted by covariates	V1 (0.227 L/kg) V2 V3	V2 alters with age k12 k21 k10 alters with LBM	Allometric scaling for volumes of distribution and clearance for weight and age	V1, V2 alter with LBM. Clearance from plasma is esterase-dependent and scales with age & LBW
Fixed parameters	All rate constants	V1 = 4.7 L V3 k13, k31	-	V3 = 5.42 L
Limitations	No age or LBM adjustments	LBM equation can overestimate doses in obese patients (James equation issue)	Not available in all TCI pumps; less familiar to some	Elderly: smaller central compartments
Patient type	Best for average, healthy adults	Good for elderly, leaner patients	Adapts for obese, elderly, pediatric	Average, healthy adults
Practical remarks	Simple and widely used; easy to implement, but based mainly on non-pregnant adults	Commonly used for effect-site targeting and smoother hypnosis control. However, it was developed in non-obstetric adult	More flexible and potentially more physiologically representative across heterogeneous populations. Nevertheless, pregnancy-specific validation remains limited	Standard remifentanil TCI model in clinical practice. Useful for rapid-onset, short-context opioid delivery, but derived from non-pregnant adults

## Data Availability

No new data were created or analyzed in this study. Data sharing is not applicable to this article.

## Abbreviations

The following abbreviations are used in this manuscript:

TIVA	Total Intravenous Anesthesia
TCI	Target-Controlled Infusion
PK	Pharmacokinetic
PBPK	Physiology-Based Pharmacokinetic
